# High-Throughput siRNA Screening to Reveal GATA-2 Upstream Transcriptional Mechanisms in Hematopoietic Cells

**DOI:** 10.1371/journal.pone.0137079

**Published:** 2015-09-01

**Authors:** Yo Saito, Tohru Fujiwara, Keiichi Ohashi, Yoko Okitsu, Noriko Fukuhara, Yasushi Onishi, Kenichi Ishizawa, Hideo Harigae

**Affiliations:** 1 Department of Hematology and Rheumatology, Tohoku University Graduate School of Medicine, Sendai, Japan; 2 Molecular Hematology/Oncology, Tohoku University Graduate School of Medicine, Sendai, Japan; 3 Department of Hematology and Cell Therapy, Yamagata University Faculty of Medicine, Yamagata, Japan; Kanazawa University, JAPAN

## Abstract

Hematopoietic stem cells can self-renew and differentiate into all blood cell types. The transcription factor GATA-2 is expressed in both hematopoietic stem and progenitor cells and is essential for cell proliferation, survival, and differentiation. Recently, evidence from studies of aplastic anemia, MonoMAC syndrome, and lung cancer has demonstrated a mechanistic link between GATA-2 and human pathophysiology. GATA-2-dependent disease processes have been extensively analyzed; however, the transcriptional mechanisms upstream of GATA-2 remain less understood. Here, we conducted high-throughput small-interfering-RNA (siRNA) library screening and showed that YN-1, a human erythroleukemia cell line, expressed high levels of GATA-2 following the activation of the hematopoietic-specific 1S promoter. As transient luciferase reporter assay in YN-1 cells revealed the highest promoter activity in the 1S promoter fused with *GATA-2* intronic enhancer (+9.9 kb/1S); therefore, we established a cell line capable of stably expressing +9.9 kb/1S-Luciferase. Subsequently, we screened 995 transcription factor genes and revealed that *CITED2* acts as a GATA-2 activator in human hematopoietic cells. These results provide novel insights into and further identify the regulatory mechanism of GATA-2.

## Introduction

Hematopoiesis is a complex process controlled by numerous transcription factors that regulate and coordinate the expression of lineage-specific genes [1]. Previous baseline studies have suggested that the GATA family of transcription factors, which act in developmental regulation, is directly involved in hematopoiesis [2–5]. GATA-1, GATA-2, and GATA-3 are known as the hematopoietic GATA factors, given their important roles in this process [1, 4–7]. Among them, GATA-2 is required for the maintenance and expansion of hematopoietic stem cells (HSCs) and/or multipotent progenitors during early hematopoiesis [5, 8–11].

To date, the mechanisms underlying GATA-2 transcription have been extensively analyzed. Two first exons/promoters of the gene, named 1S and 1G, have been identified in both mice and humans [12, 13]. Transcripts involving the 1G promoter are commonly found in tissues expressing GATA-2, whereas 1S transcripts are believed to play an important role in hematopoietic cells [12, 13]. During erythroid differentiation, GATA-2 levels decline concomitantly with an increase in GATA-1 levels [5]. GATA-1 represses *Gata2* transcription by displacing GATA-2 from the sites at −77, −3.9, −2.8, −1.8, and +9.5 kilobase (kb) relative to the 1S promoter, which are known as GATA switch sites [5, 14]. However, despite the compelling evidence supporting the locations and characteristics of these GATA switch sites, targeted individual deletions of the −1.8, −2.8, and −3.9 kb sites lead to minor increases in *Gata2* expression in murine hematopoietic precursors [15–17]. On the other hand, deletion of the +9.5 site leads to delayed embryonic lethality compared with global *Gata2* knockout [18]. Noticeably, in humans, the heterozygous mutation of the intronic enhancer at +9.9 kb, which corresponds to +9.5 kb in mice, has been found in patients with GATA-2 deficiency (MonoMAC syndrome) [18], which is characterized by a predisposition to myelodysplastic syndrome (MDS) and acute myeloid leukemia (AML) [19–21]. Thus, the 1S promoter and +9.5 kb enhancer regions could be considered as important regulatory regions for *GATA-2* expression.

Several transcription factors involved in various signaling pathways, such as the Wnt and Notch pathways, are known to participate in GATA-2 regulation [22, 23]; however, relatively less is known about how these transcriptional molecular mechanism associate with GATA-2 expression. Given the pathophysiological links between GATA-2 and aplastic anemia, MonoMAC syndrome, and lung cancer [19–21, 24–26], it is extremely important to clarify and comprehensively understand the details regarding the mechanisms behind the upstream transcription of *GATA-2*. With this in mind, we conducted a high-throughput screening analysis based on a small interfering RNA (siRNA) library to provide a novel insights into the factors involved in the regulation of GATA-2 expression.

## Materials and Methods

### Cell culture

The human erythroleukemia cell lines YN-1 [27] and K562 [28], human myeloid leukemia cell line KG1a [29], human T cell leukemia cell line Jurkat, human monocytoid cell line U937, and the human pre-B cell leukemia cell line NALM6 were cultured in RPMI-1640 (Sigma-Aldrich, St. Louis, MO, USA) supplemented with 10% fetal bovine serum (FBS; Biowest, Miami, FL, USA) and 1% penicillin/streptomycin (Sigma-Aldrich). Stable YN-1 cells expressing pGL4.20 (*GATA-2* +9.9/1S; described below) were cultured in RPMI-1640 containing 10% FBS, 1% penicillin/streptomycin, and 1 μg/ml puromycin (Sigma-Aldrich). K562 cells were obtained from American Type Culture Collection (ATCC, Manassas, VA, USA); other cell lines (YN-1, KG1a, Jurkat, U937, and NALM6) were obtained from the Cell Resource Center for Biomedical Research at Tohoku University (www2.idac.tohoku.ac.jp/dep/ccr//).

### Plasmids

*GATA-2* sequences were cloned from bacterial artificial chromosome DNA (RP11-475N22: Empire Genomics, Buffalo, NY, USA). Primers linked to restriction enzyme sites were used to amplify the *GATA-2* genomic region to be included in the plasmids (primer sequences are available upon request). DNA sequence analysis was used to confirm the integrity of the cloned sequences. The luciferase reporter vectors, pGL3 (Luc) and pGL4.20 (Luc2/puro) and renilla vectors pRL and pGL4.74 were purchased from Promega (Madison, WI, USA). All restriction enzymes described below were purchased from Toyobo (Osaka, Japan).

The 1S-Luc construct was created by amplifying a 500-base pair (bp) sequence of the *GATA-2* 1S promoter region using the appropriate primer pair (S1 Table). This sequenced region was subsequently digested with MluI and BglII, and the digested products were purified and ligated into the pGL3 basic vector, which had been previously digested and purified using the same pair of restriction enzymes. The (−1)Luc and (−1.8)Luc plasmids were created using the NheI and BglII restriction enzymes by the same protocol used for 1SLuc. (−3.4)Luc, (−4.6)Luc, and (−4.6)Luc2 and using GeneArt SeamLess cloning (Invitrogen, Carlsbad, CA, USA). The *GATA-2* +9.9 kb site was inserted upstream of the 1SLuc, (−3.4)Luc, or (−4.6)Luc sequence. GATA and/or E-box mutated constructs were generated using a QuickChange™ Site-Directed Mutagenesis Kit (Agilent technologies, Santa Clara, CA, USA).

For GATA-2 overexpression, *GATA-2* mRNA was cloned into the pBABE-puro vector (Addgene Plasmid 1764; Addgene Cambridge, MA, USA).

### Promoter assay

To evaluate the *GATA-2* transcriptional activity, aliquots of YN-1 cells were transfected with 1 μg of *GATA-2* promoter construct and 100 ng of the pRL or the pGL4.74 [*hRluc*/TK] vector (Promega) via FuGene HD (Promega). The cells were harvested 24 h after plasmid transfection, and firefly and *Renilla* luciferase activity levels in the cell extracts were determined using a Dual-Luciferase Reporter Assay System (Promega) or a ONE-Glo Luciferase Assay System, with GloMax 20/20 Luminometer (Promega).

### Retroviral gene transfer

Retroviral GATA-2 expression was conducted as described previously [30].

### siRNA screening

For siRNA screening, we targeted 995 genes encoding transcription factors from the siPerfect transcription factor library (RNAi, Tokyo, Japan). The library was arranged in 13 96-well plates (Thermo Scientific, Yokohama, Japan), with each well containing 5 pmol of a pool of 21-mer siRNA duplexes. Lamin A siRNA and nontarget siRNA were used as the positive and negative controls, respectively. Next, 1 × 10^4^ stable YN-1 cells expressing pGL4.20 (*GATA-2* +9.9/1S) in 100 μl of RPMI containing puromycin were transferred to each of the 96 wells in each plate (Berthold, Land Baden-Württemberg, Germany). We added the transfection reagent GenomONE-Si (Ishihara Sangyo, Osaka, Japan) to the siRNA library plates according to the manufacturer’s protocol. Subsequently, we added 0.9 μl of siRNA transfection mixture per well (final concentration in wells, 5nM). siRNA transfection was conducted twice (at 0 and 24 h). At 48 h, we assessed the luciferase activity of each well using a ONE-Glo Luciferase Assay System (Promega) and Berthold Centro LB960 luminometer (Berthold). For the luciferase activity assay, we added 100 μl of ONE-Glo Reagent to each well on the plates and evaluated firefly luciferase activity with a luminometer after incubating the plates for 3 min at room temperature.

### siRNA-mediated knockdown

For siRNA-mediated knockdown, 1 × 10^6^ YN-1 cells with stable expression of pGL4.20 (*GATA-2* +9.9/1S) were transfected with an Amaxa Nucleofector (Nucleofector solution V, Nucleofector program T-016; Lonza Group, Basel, Switzerland). The antisense siRNA sequences specific for human Brahma related gene 1 (*BRG1*) were: GCACACCGCUGCAGAACAA, CCAAGCCGGUCGUGAGUGA, GCGACUCACUGACGGAGAA, and GACCAGCACUCCCAAGGUU. For human CBP/p300-Interacting Transactivator with Glu/Asp-Rich C-Terminal Domain, 2 (*CITED2*) knockdown, we used the sequences GUUCUUAUGUCCUUGGUGA, CAACCAGUAUUUCAACCAU, GAAAUGGGUUUGGACCGCA, and CUGCAGGCCACCAGAUGAA (Dharmacon, Lafayette, CO, USA). The siGENOME non-targeting siRNA pool #1 (Thermo Scientific Dharmacon) was used as a negative control. siRNA sequences specific for *GATA-2* knockdown and the corresponding negative control were as described previously [31].

### CRISPR/Cas9-based genome editing

To knockout endogenous CITED2 in YN-1 cells, the GeneArt CRISPR Nuclease Vector with CD4 enrichment kit (GE Life Sciences/Dharmacon) was used. The target sequence for nuclease recruitment was CATATGATGGCCATGAACCACGG (+25 to +47 from the translation initiation site, according to NCBI accession number NM_001168389). The plasmid was transfected using an Amaxa Nucleofector (Nucleofector solution V, Nucleofector program T-016; Lonza Group), and CD4-positive cells were subsequently cloned.

### Real-time quantitative reverse transcriptase (RT)-polymerase chain reaction (PCR)

Real-time quantitative RT-PCR was conducted using the SYBR Green master mix (Qiagen, Venlo, The Netherlands) according to a previously described protocol [32]. All primer sequences are listed in S1 Table.

### Western blotting analysis

Whole-cell lysates were prepared by boiling 1 × 10^7^ cells/ml in sodium dodecyl sulfate (SDS) buffer [32]. Samples (5 μl) were resolved using SDS-polyacrylamide gel electrophoresis and analyzed with specific antibodies.

### Antibodies

Antibodies specific for GATA-2 (H-116, sc-9008) and CITED2 (JA22, sc-21795) were purchased from Santa Cruz Biotechnology (Santa Cruz, CA, USA). A specific alpha-tubulin antibody was purchased from Calbiochem (Darmstadt, Germany). CITED2-specific antibody (EPR3416, ab108345) and rabbit control IgG were purchased from abcam (Cambridge, MA, USA).

### Statistics

Statistical significance was assessed with a two-sided Student’s *t* test.

## Results and Discussion

### *GATA-2* 1S transcript is highly expressed in the YN-1 cell line

**Fig 1:**
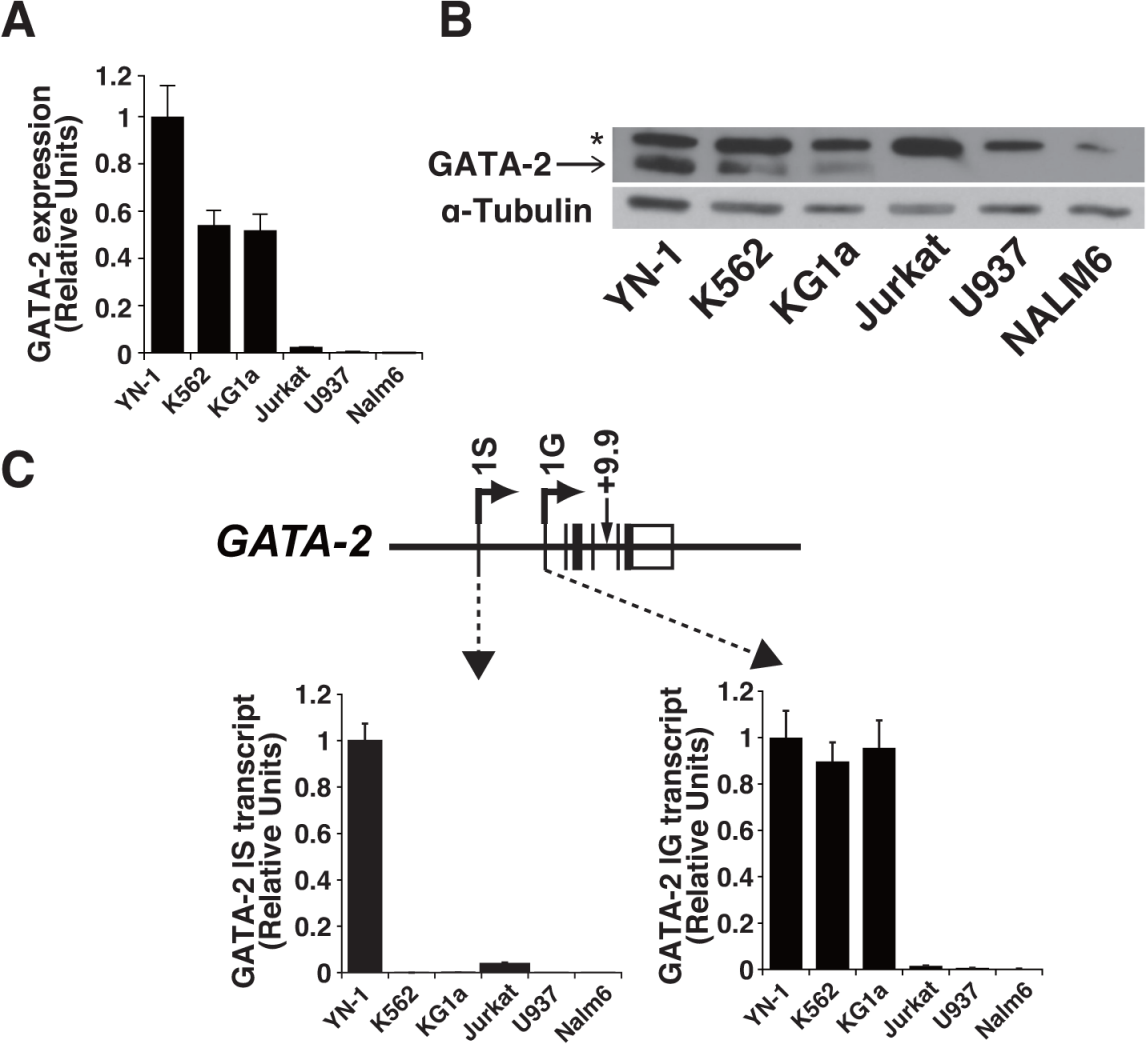
*GATA-2* 1S expression in YN-1 cells. Quantitative reverse transcriptase-polymerase chain reaction (RT-PCR) and western blotting were performed to measure GATA-2 expression in six hematopoietic cell lines. (A) Quantitative RT-PCR for *GATA-2* mRNA (mean ± standard error [SE], n = 3). GAPDH mRNA was used as a control. The *GATA-2* mRNA expression level in YN-1 cells was set to 1. (B) Anti-GATA-2 western blotting analysis of whole-cell extracts from six cell lines. Alpha-tubulin was used as a loading control. The asterisk demotes cross-reactive band. (C) Quantitative RT-PCR for *GATA-2* 1S or 1G mRNA (mean ± SE, n = 3). *GAPDH* mRNA was used as a control. The *GATA-2* *1S* and *1G* mRNA expression levels in YN-1 cells were set to 1.

To conduct high-throughput siRNA screening, the selection of a hematopoietic cell line that expresses high levels of the target gene, in this case *GATA-2*, is desirable. In this study, we selected six hematopoietic cell lines, YN-1, K562, KG1a, Jurkat, U937, and NALM6, as candidate lines. Real-time quantitative RT-PCR and western blot analyses revealed that GATA-2 was abundantly expressed on both the gene and protein levels in YN-1, K562, and KG1a cell lines, but was almost undetectable in Jurkat, U937, and Nalm6 cells (Fig 1A and 1B). The validity of the GATA-2 antibody was confirmed by both GATA-2 overexpression and knockdown in K562 cells (S1 Fig). As previously described, GATA-2 transcription involves two different exons, a distal (1S) and a proximal (1G) promoter; the former is considered to be particularly important in hematopoietic cells [5, 12, 13], although it remained unknown regarding the contribution of 1S and 1G transcripts to the absolute level of GATA-2 expression in hematopoietic cells. Therefore, we conducted a quantitative RT-PCR analysis of each transcript. The *GATA-2* 1G transcript was expressed in YN-1, K562, and KG1a cells (Fig 1C); noticeably, GATA-2 1S was the most abundantly expressed in YN-1 cells (Fig 1C). KG1a cells were derived from a human patient who presented with acute myelogenous leukemia characterized by an undifferentiated blast cell phenotype [29]. Therefore, the cells might have retained the characteristics of HSCs and thereby expressed GATA-2. On the other hand, the K562 and YN-1 lines were derived from a patient with chronic myelogenous leukemia [27, 28]. Although GATA-2 expression has already been confirmed in K562 cells [28, 32], the relative expression levels of 1S and 1G transcript had not yet been characterized. In addition, GATA-2 expression had not been evaluated in YN-1 cells, and the cause of the abundant expression of *GATA-2* 1S in this cell line remained unknown. In the current study, we selected the YN-1 cell line for siRNA screening.

### *GATA-2* 1S promoter fused to +9.9 kb enhancer induced high promoter activity

**Fig 2:**
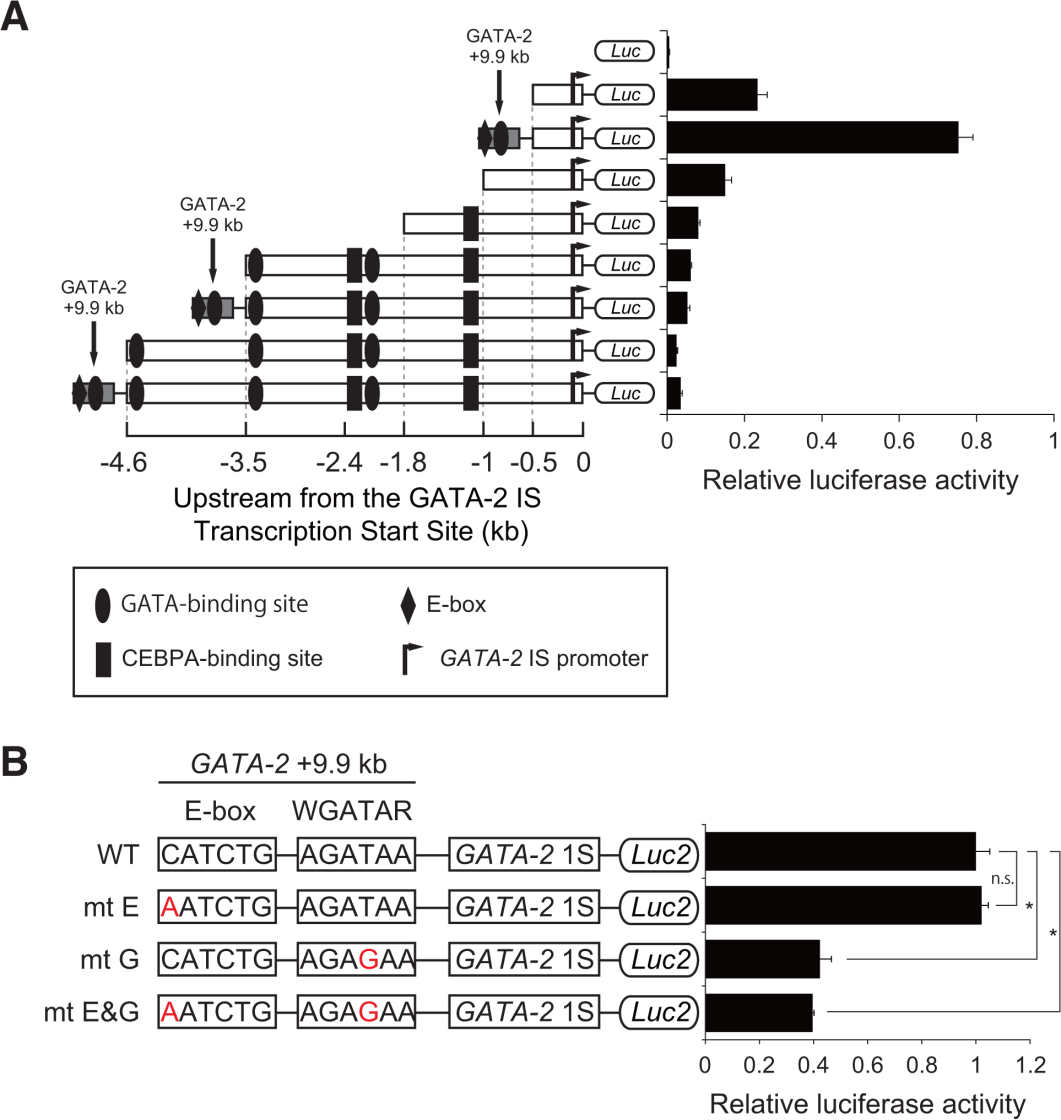
*GATA-2* 1S promoter activity is associated with the +9.9 kb enhancer. (A) The enhancer activities of the constructs, which included *GATA-2* upstream or intron regions with various combinations of *GATA* binding sites, CCAAT/enhancer binding protein, alpha (*CEBPA*) binding sites, and an E-box. YN-1 cells were transiently cotransfected with Renilla (pRL) and luciferase vectors (Luc). The luciferase vector was either empty or contained the *GATA-2 1S* promoter cloned upstream of luciferase (1SLuc), with or without the upstream 1S promoter sequence and/or +9.9 kb site. Luciferase activity was measured 24 h after the first transfection. Results are presented as ratios of luciferase activities (means of Firefly/Renilla ± standard error, n = 3). (B) Impact of the GATA-binding site and/or E-box site mutation on the +9.9/1S promoter activity. We generated wild-type, E-box only, GATA-binding site only and double-mutation constructs fused to a luciferase reporter gene and performed a transient transfection assay in YN-1 cells (mean ± standard deviation, n = 3). * p<0.05.

We conducted a transient luciferase promoter analysis to determine the optimal configuration for siRNA screening. Based on the importance of 1S promoter for GATA-2 expression in hematopoietic cells [12, 13], we selected 1S promoter as a potential promising tool to identify novel upstream factor of GATA-2 in hematopoietic cells. As shown in Fig 2A, the 1S promoter exhibited a modest promoter activity, and the addition of a +9.9 kb enhancer to this promoter (+9.9/1S) resulted in a higher luciferase activity. On the other hand, addition of -4.6 to -0.5 kb fragment to the 1S promoter led to lower promoter activity compared with that of the 1S promoter alone (Fig 2A). The reason for lower promoter activity following addition of −4.6 to −1 kb sequence could be partially explained by the repressive effect of CEBPA (CCAAT/enhancer binding protein, alpha) through its binding site at −1.2 and −2.4 kb sites (Fig 2A) [33]. Low promoter activity based on a sequence alteration at −1 kb, which does not include CEBPA binding sites (Fig 2A), might imply the presence of other unrecognized repressive elements at this position. Regarding the functional validation of the sequence +9.9/1S, we introduced a mutation within the GATA-binding element and/or E-box motifs and demonstrated that the GATA-binding domain plays an important role in luciferase activity induced by the +9.9/1S construct (Fig 2B). Wozniak et al. [34] analyzed the murine +9.5/1S construct in detail; these authors demonstrated that the +9.5/1S construct showed a high luciferase activity, and the disruption of the GATA binding element within +9.5 kb eliminated its enhancer activity. Therefore, we selected the +9.9 kb/1S-luciferase construct for the screening.

### Establishment of YN-1 cells with stable luciferase activity under the control of +9.9 kb/1S

**Fig 3:**
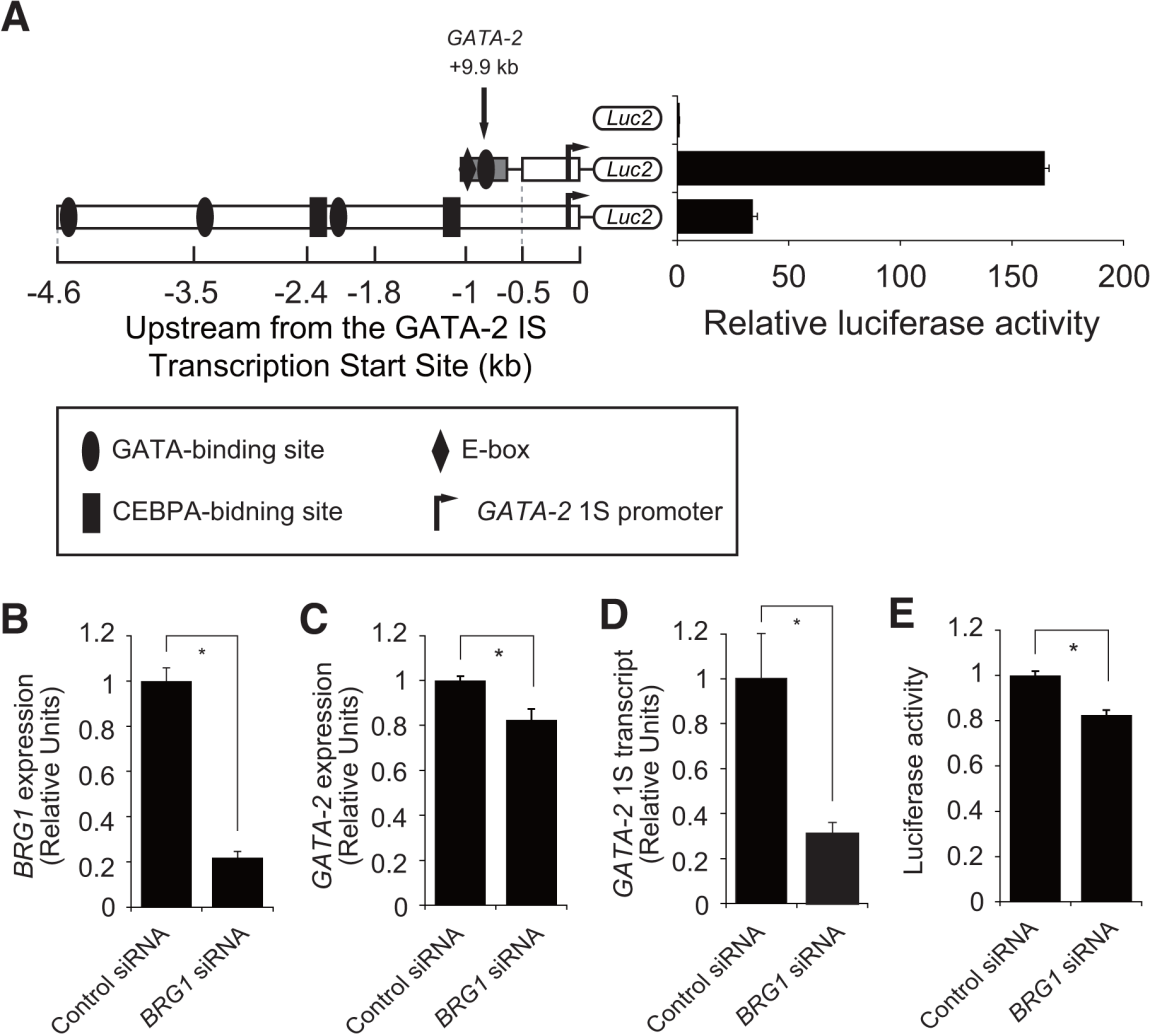
Establishment of YN-1 cells showing stable luciferase activity. (A) YN-1 cells were transfected with pGL4.20 (GATA-2 +9.9/1S or -4.6) and selected using puromycin. (B) Small-interfering-RNA (siRNA)-mediated knockdown of Brahma related gene 1 (*BRG1*) was conducted in YN-1 cells stably expressing luciferase activity under the control of +9.9/1S. Transfection was conducted twice via electroporation, at 24-h intervals. The cells were harvested for assays 48 h after the first transfection, and quantitative reverse transcriptase-polymerase chain reaction for Brahma related gene 1 (BRG1) was conducted (mean ± standard error [SE], n = 3, p < 0.001). (C-E) *BRG1* knockdown resulted in the significant downregulation of both *GATA-2* expression (mean ± SE, n = 3, p < 0.001) (C), *GATA-2* 1S transcript (mean ± SE, n = 3, p < 0.001) (D), and luciferase activity (mean ± SE, n = 3, p = 0.0044) (E). * p<0.05.

A luciferase plasmid was introduced into the YN-1 cells, which were subsequently selected using puromycin to ensure stable expression. We used an empty luciferase vector (Luc2) and a vector containing a +9.9/1S or −4.6 kb sequence. Similar to the results of the transient luciferase assay (Fig 2), the luciferase activity was highest in the YN-1 cells expressing the +9.9 kb/1S construct (Fig 3A). Next, to test whether an already known GATA-2 regulator might affect the luciferase activity, we knocked down *BRG1* [15]. Previous reports have shown that *Brg1* knockdown in G1E cells repressed the expression of GATA-2, through its effect on the +9.5 enhancer [15]. As shown in Fig 3B–3E, knockdown of *BRG1* resulted in a significant downregulation of *GATA-2* expression and luciferase activity.

### High-throughput siRNA screening identified *GATA-2* transcription regulators

**Fig 4:**
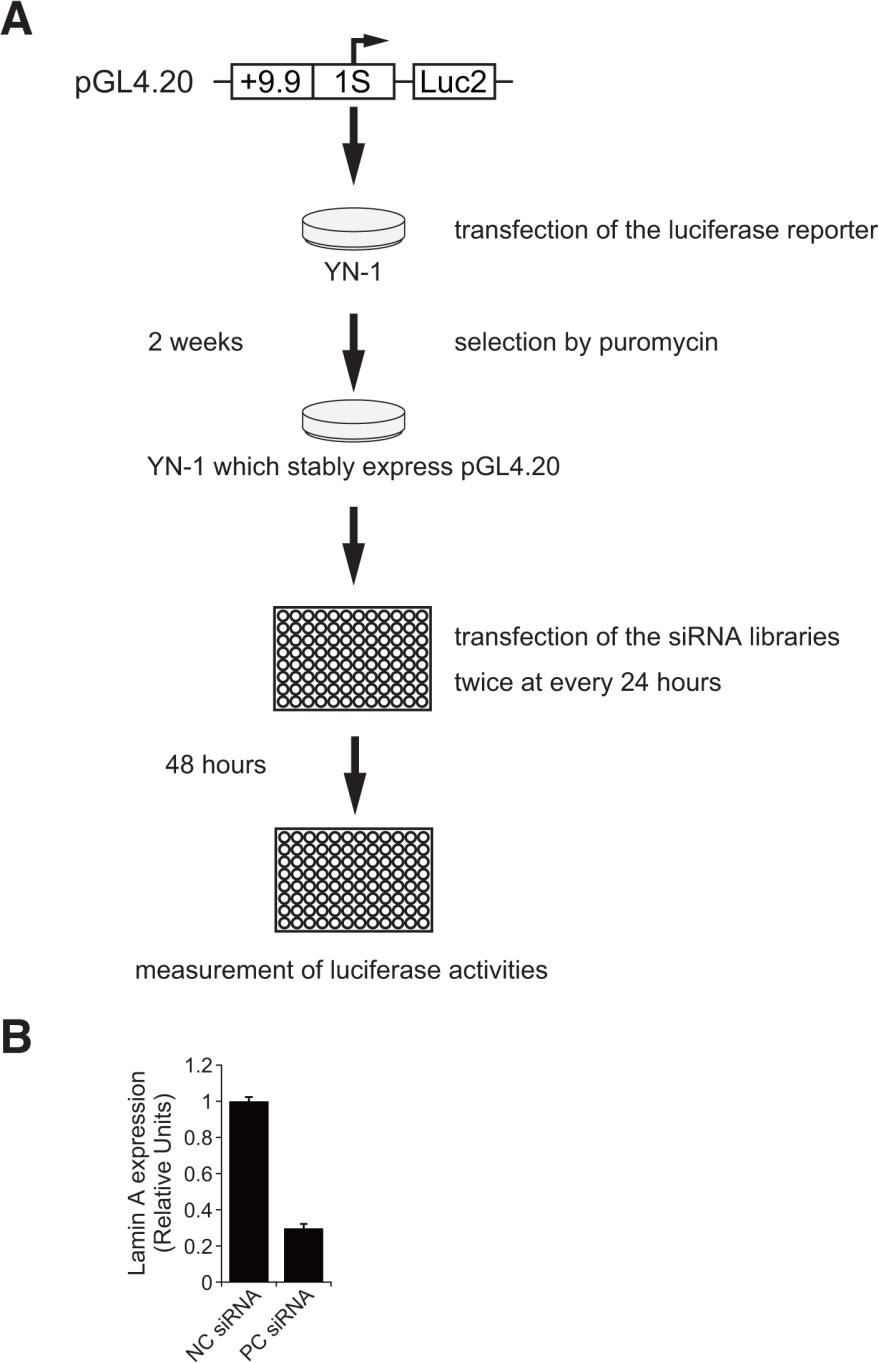
High-throughput small interfering RNA (siRNA) screening to identify regulators of *GATA-2* transcription. (A) Schematic representation of the high-throughput siRNA screening. YN-1 cells with stable +9.9/1S-*Luc2* expression were selected using puromycin. Subsequently, the cells were transfected twice at 24-h intervals. Luciferase activity was assessed 48 h after the first transfection. We used an siPerfect siRNA library (RNAi) that targeted 995 transcription factor genes. (B) We used *Lamin A* siRNA to validate the efficiency of the transfection protocol (mean ± standard error, n = 3, p < 0.001). * p<0.05.

To discover novel regulators of GATA-2 expression, we conducted siRNA-based high-throughput screening. As shown in Fig 4A, YN-1 cells expressing +9.9 kb/1S-Luc2 were transfected twice with siRNA at 24-h intervals, and luciferase activity was assessed after 48 h. We used an siRNA library that contained a total 995 genes involved in transcriptional regulation. To test whether our transfection protocol could efficiently knockdown the target genes, siRNA directed against Lamin A was included as a positive control, and knockdown of the encoding gene was confirmed via quantitative RT-PCR (Fig 4B).

Following the screening analysis, the top 10% of the genes that significantly (p < 0.05) repressed luciferase activity after their knockdown were selected from each plate (n = 83: Table 1, S2 Table), which included potential GATA-2 regulators of GATA-1 and GFI1B. On the other hand, however, the analysis did not identify known GATA-2 regulators, such as TAL1 [32, 35], presumably due to the limited screening sensitivity. Subsequently, we narrowed down the genes, based on their potential importance in hematopoiesis. In the present study, we focused on the gene CITED2 (Cbp/p300-interacting transactivator, with Glu/Asp-rich carboxy-terminal domain, 2).

**Table 1:** Candidates of GATA-2 activators.

Plate No.	Symbol	Fold change	SE	p-value	Plate No.	Symbol	Fold change	SE	p-value
	MDM2	0.73	0.14	0.006		CITED2	0.39	0.03	< 0.001
	DDX54	0.74	0.11	0.004		CNOT7	0.40	0.03	< 0.001
1	HEXIM1	0.77	0.14	0.013		BRDT	0.41	0.01	< 0.001
	CXXC1	0.79	0.10	0.009	7	CNOT2	0.42	0.09	< 0.001
	MED12	0.79	0.11	0.012		CITED1	0.42	0.01	< 0.001
	MIXL1	0.74	0.05	< 0.001		BRCA1	0.43	0.04	< 0.001
	ESRRB	0.78	0.11	0.007		EGR1	0.43	0.04	< 0.001
	MED31	0.78	0.07	0.002		DLX3	0.43	0.06	< 0.001
2	HOP	0.78	0.12	0.009		FOXD1	0.69	0.09	0.014
	EN1	0.80	0.03	0.001	8	GATA1	0.71	0.13	0.025
	ERG	0.82	0.04	0.004		GFI1B	0.75	0.06	0.035
	HLX1	0.83	0.09	0.018		KLF11	0.57	0.09	< 0.001
	LEF1	0.83	0.06	0.009		MYOD1	0.60	0.05	< 0.001
	MYT1L	0.61	0.11	< 0.001		KLF5	0.63	0.13	< 0.001
	RARG	0.71	0.02	< 0.001	9	MLL	0.64	0.04	< 0.001
	MYST2	0.71	0.18	0.006		ISGF3G	0.64	0.04	< 0.001
3	NKX2-2	0.71	0.03	< 0.001		NAB2	0.65	0.06	< 0.001
	NPAS3	0.73	0.04	< 0.001		NEUROG1	0.65	0.06	< 0.001
	NR1H2	0.74	0.10	< 0.001		FTHL8	0.68	0.09	< 0.001
	PKNOX2	0.75	0.07	< 0.001		PLAG1	0.59	0.06	< 0.001
	MTA3	0.75	0.21	0.025		PTTG1	0.60	0.05	< 0.001
	NFY	0.57	0.08	< 0.001		NFRKB	0.61	0.01	< 0.001
	VDR	0.59	0.04	< 0.001	10	POUF2	0.63	0.08	< 0.001
	PPARD	0.59	0.05	< 0.001		PIAS1	0.64	0.06	< 0.001
4	ZFHX1B	0.60	0.09	0.002		NR3C1	0.64	0.06	< 0.001
	CBFA2T2	0.60	0.08	0.001		POU2F1	0.64	0.09	< 0.001
	ZNF694	0.60	0.05	0.001		POU6F1	0.64	0.05	< 0.001
	NKX2-5	0.61	0.04	0.001		TBX4	0.62	0.04	< 0.001
	ZFHX4	0.61	0.02	0.001		TCEA1	0.71	0.09	< 0.001
	FOXF2	0.55	0.03	< 0.001		TBX22	0.71	0.02	< 0.001
	HEYL	0.63	0.13	< 0.001	12	TSFM	0.71	0.01	< 0.001
	GTF2IRD1	0.67	0.16	0.001		TCF8	0.73	0.18	0.016
5	ELF4	0.73	0.08	< 0.001		TCEB3B	0.75	0.02	< 0.001
	FOXF1	0.76	0.09	< 0.001		TBX3	0.76	0.04	0.001
	FOXA2	0.77	0.07	< 0.001		TCFL5	0.80	0.03	0.003
	HCFC1	0.77	0.17	0.018		ZNF140	0.64	0.04	< 0.001
	MEF2D	0.78	0.13	0.007	13	NZF161	0.67	0.13	< 0.001
	TFE3	0.74	0.16	0.018		ZNF35	0.67	0.02	< 0.001
	T	0.76	0.00	0.002		ZIC1	0.67	0.11	< 0.001
	TCEB3	0.76	0.08	0.005					
6	ZNF398	0.77	0.07	0.005					
	VSX1	0.77	0.11	0.012					
	ARID4A	0.78	0.06	0.006					
	WT1	0.85	0.04	0.035					

Fold change represents mean luciferase activity of each sample relative to that of negative control (n = 3).

**Fig 5:**
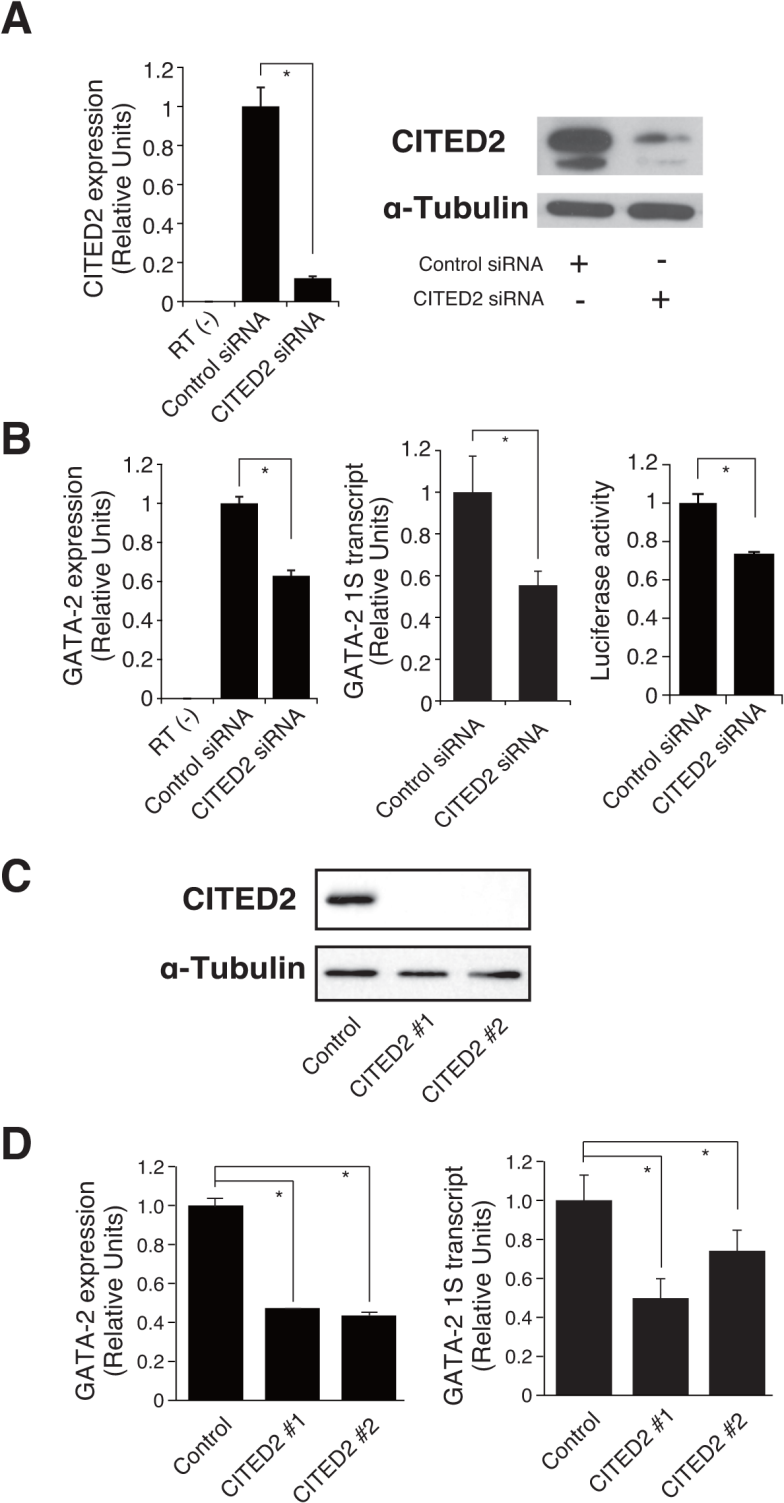
Identification of CBP/p300-Interacting Transactivator with Glu/Asp-Rich C-Terminal Domain 2 (CITED2) as an activator of GATA-2 expression activators. (A, B) *CITED2* was knocked down with a pool of four synthetic small interfering RNAs (siRNAs) (Dharmacon); this gene was targeted in YN-1 cells expressing +9.9 kb/1S-Luc2 to validate the screening analysis. The knockdown process was repeated twice at 24-h intervals. The cells were harvested and assayed 48 h after the first transfection. (A) *CITED2* mRNA was significantly repressed, by 12%, after siRNA transfection (mean ± standard error [SE], n = 3, p < 0.001), and this result was confirmed by western blotting. Alpha-tubulin was used as a loading control. (B) *GATA-2* mRNA expression, *GATA-2* 1S transcript, and luciferase activity decreased by 63% (mean ± SE, n = 3, p < 0.001), 55% (mean ± SE, n = 3, p < 0.001), and 74% (mean ± SE, n = 3, p < 0.001), respectively. (C, D) CITED2 knockout clones (#1, #2), established from YN-1 cells, and confirmed by western blotting (C). In these clones, *GATA-2* mRNA expression and *GATA-2* 1S transcript were significantly decreased (mean ± SE, n = 3, p < 0.001) (D). * p<0.05.

**Fig 6:**
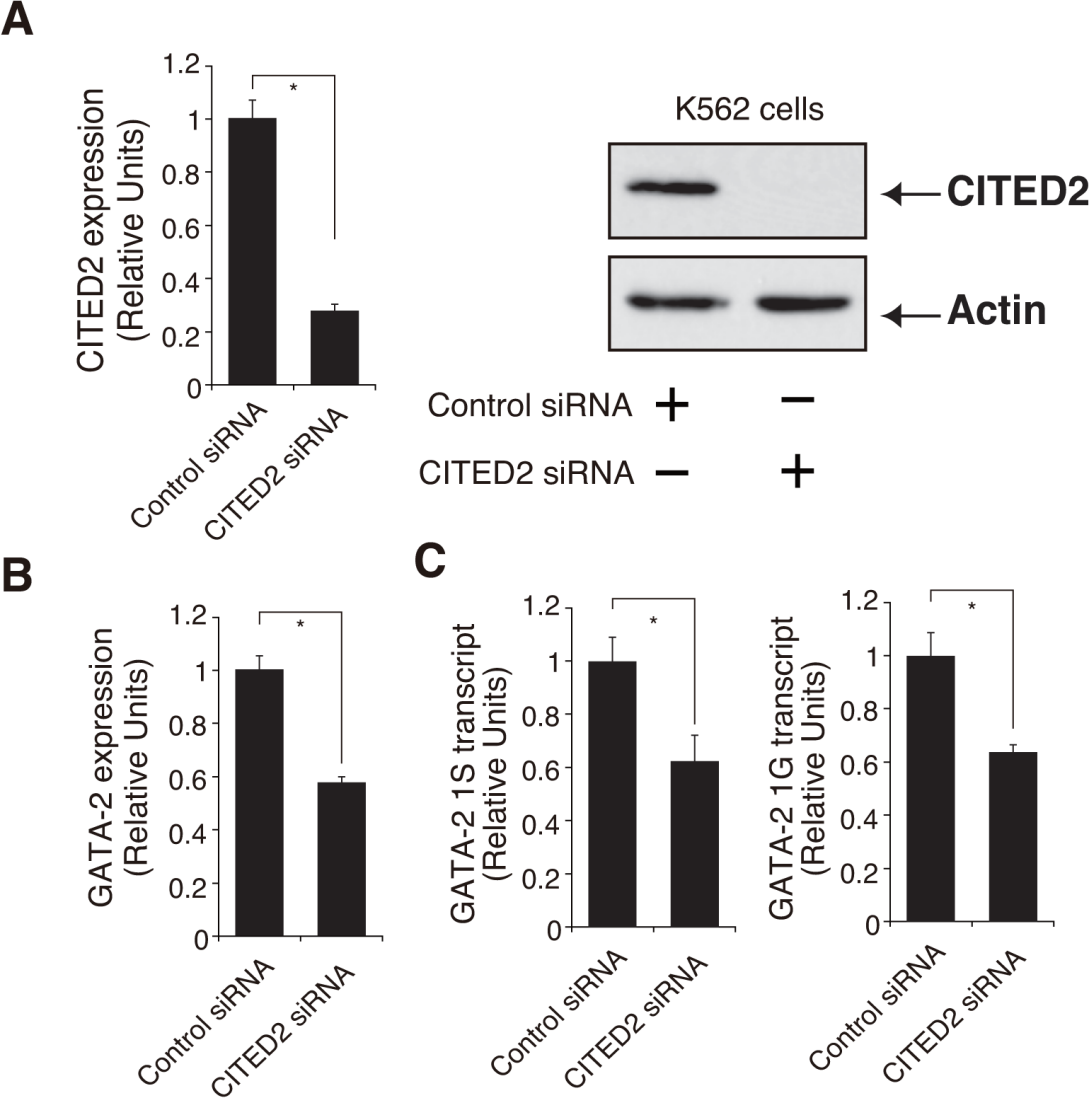
*CBP/p300-Interacting Transactivator with Glu/Asp-Rich C-Terminal Domain 2* (CITED2) regulates GATA-2 expression in K562 cells. (A) *CITED2* was knocked down with a pool of four synthetic small interfering RNAs (siRNAs) (Dharmacon) in K562 cells. The knockdown process was repeated twice at 24-h intervals. The cells were harvested and assayed 48 h after the first transfection. (A) *CITED2* mRNA was significantly repressed after siRNA transfection (mean ± standard error [SE], n = 3, p < 0.001), and this result was confirmed by western blotting. Actin was used as a loading control. (B,C) *GATA-2* mRNA expression and *GATA-2* 1S and 1G transcripts were significantly decreased (mean ± SE, n = 3, p < 0.001). * p<0.05.

We next knocked down *CITED2* using a pool of four synthetic siRNAs based on YN-1 cells expressing +9.9 kb/1S-Luc2. As shown in Fig 5A, the expression of *CITED2* genes was significantly repressed (by 12%) after siRNA transfection, a result that was confirmed by western blot analysis. Accordingly, we confirmed that *GATA-2* mRNA expression, GATA-2 1S transcript, and luciferase activity significantly decreased (by 63%, 55%, and 74%, respectively), under these conditions (Fig 5B). We further established two CITED2-knockout YN-1 cell clones based on CRISPR/Cas9 technique and confirmed these clones by western blotting (Fig 5C). Quantitative RT-PCR analysis confirmed significant downregulation of *GATA-2* mRNA and 1S transcript (Fig 5D). To reveal how CITED2 contributes to the GATA-2 expression in general, we also performed siRNA-mediated CITED2 knockdown based on K562 cells, which exhibited relatively lower 1S transcript level than YN-1 cells (Fig 1C). As shown in Fig 6, CITED2 knockdown in K562 cells resulted in significant downregulation of total GATA-2 mRNA as well as both 1S and 1G transcript levels, suggesting that CITED2 might not specifically activate +9.9 kb/1S promoter. Furthermore, to test if CITED2 would specifically affect +9.9 kb enhancer activity, luciferase analysis was conducted based on YN-1 cells and CITED2-knockout YN-1 cell clone (#1), demonstrating that CITED2 depletion significantly reduced both 1S alone and +9.9kb/1S-luciferase activity (S2 Fig). Thus, CITED2 might also regulate GATA-2 expression by impacting upon promoter region. Whereas our results indicated that CITED2 is an upstream regulator of GATA-2 in hematopoietic cells, further analysis would be required to reveal the molecular mechanism by which CITED2 contributes to the GATA-2 expression.

CITED2 is known as CBP/p300-dependent transcriptional co-activator [36–38]. In a previous study, conditional deletion of *Cited2* in adult mice resulted a loss of HSCs, leading to bone marrow failure and increased lethality [36]. Noticeably, in *Cited2* ^−/−^ Lin^−^c-Kit^+^ cells exhibited reduced *Bmi1*, *Notch1*, *Lef1*, *Mcl1*, and *Gata2* expression [37]. Furthermore, a previous report demonstrated that BMI1 could induce *Gata2* expression [39]. Based on an analysis of clinical samples from patients with AML, Korthuis *et al*. recently demonstrated that CITED2 might play an important role in maintenance of this disease [40]. These data support our finding that *CITED2* is an upstream regulator of GATA-2 in humans.

Aplastic anemia (AA) is characterized by a decrease in HSCs and fatty marrow replacement. We, along with other research groups, have demonstrated reduced GATA-2 expression in CD34-positive cells from patients with AA [24, 25]. On the other hand, decreased GATA-2 expression in mesenchymal stem cells has been shown to lead to accelerated adipocyte differentiation and an impaired ability to support hematopoiesis [41]. Therefore, in the bone marrow, GATA-2 not only generates and/or maintains HSCs, but also plays a role in the regulating the hematopoietic microenvironment. Thus, our system potentially represents a powerful tool with which to identify the regulatory mechanisms of GATA-2 and might lead to the development of novel therapeutic approaches for bone marrow failure syndrome.

## Supporting Information

S1 Fig Identification of GATA-2 by western blot. Western blot analysis to detect GATA-2 in K562 cells. To specify GATA-2 band, both overexpression and knockdown of GATA-2 were conducted in K562 cells. Alpha-tubulin was used as a loading control. The asterisk demotes cross-reactive band.
(EPS)

S2 Fig *CBP/p300-Interacting Transactivator with Glu/Asp-Rich C-Terminal Domain 2* (CITED2) affects both 1S and +9.9 kb/1S luciferase activity. Luciferase analysis was performed with empty or contained the *GATA-2 1S* promoter and/or +9.9 kb site in both YN-1 cells and CITED2-knockout YN-1 clone (Fig 5). Luciferase activity was measured 24 h after the first transfection. Results are presented as ratios of luciferase activities (means of Firefly/Renilla ± standard error, n = 3).* p<0.05.
(EPS)

S1 Table Oligonucleotide primers. (EPS)

S2 Table Results of *small interfering RNA (siRNA) screening*. (XLSX)
